# Development and Validation of a Novel Prognostic Nomogram Combined With Desmoplastic Reaction for Synchronous Colorectal Peritoneal Metastasis

**DOI:** 10.3389/fonc.2022.826830

**Published:** 2022-03-11

**Authors:** Xiusen Qin, Mingpeng Zhao, Weihao Deng, Yan Huang, Zhiqiang Cheng, Jacqueline Pui Wah Chung, Xufei Chen, Keli Yang, David Yiu Leung Chan, Hui Wang

**Affiliations:** ^1^ Department of Colorectal Surgery, The Sixth Affiliated Hospital of Sun Yat-sen University, Guangzhou, China; ^2^ Guangdong Institute of Gastroenterology, Guangdong Provincial Key Laboratory of Colorectal and Pelvic Floor Diseases, Supported by the National Key Clinical Discipline, The Sixth Affiliated Hospital of Sun Yat-sen University, Guangzhou, China; ^3^ Assisted Reproductive Technology Unit, Department of Obstetrics and Gynaecology, Faculty of Medicine, Chinese University of Hong Kong, Hong Kong, Hong Kong SAR, China; ^4^ Department of Pathology, The Sixth Affiliated Hospital of Sun Yat-sen University, Guangzhou, China; ^5^ Department of Obstetrics and Gynaecology, Songshan Lake Central Hospital, Affiliated Dongguan Shilong People’s Hospital of Southern Medical University, Dongguan, China

**Keywords:** colorectal cancer, synchronous peritoneal metastasis, prognosis, desmoplastic reaction, cancer-associated fibroblasts

## Abstract

**Purpose:**

The prognostic value of desmoplastic reaction (DR) has not been investigated in colorectal cancer (CRC) patients with synchronous peritoneal metastasis (SPM). The present study aimed to identify whether DR can predict overall survival (OS) and develop a novel prognostic nomogram.

**Methods:**

CRC patients with SPM were enrolled from a single center between July 2007 and July 2019. DR patterns in primary tumors were classified as mature, intermediate, or immature according to the existence and absence of keloid-like collagen or myxoid stroma. Cox regression analysis was used to identify independent factors associated with OS and a nomogram was developed subsequently.

**Results:**

One hundred ninety-eight and 99 patients were randomly allocated into the training and validation groups. The median OS in the training group was 36, 25, and 12 months in mature, intermediate, and immature DR categories, respectively. Age, T stage, extraperitoneal metastasis, differentiation, cytoreductive surgery (CRS), hyperthermic intraperitoneal chemotherapy (HIPEC), and DR categorization were independent variables for OS, based on which the nomogram was developed. The C-index of the nomogram in the training and validation groups was 0.773 (95% CI 0.734–0.812) and 0.767 (95% CI 0.708–0.826). The calibration plots showed satisfactory agreement between the actual outcome and nomogram-predicted OS probabilities in the training and validation cohorts.

**Conclusions:**

DR classification in the primary tumor is a potential prognostic index for CRC patients with SPM. The novel prognostic nomogram combined with DR classification has good discrimination and accuracy in predicting the OS for CRC patients with SPM.

## Introduction

Peritoneal metastasis (PM) occurs in 5%–10% of newly diagnosed colorectal cancer (CRC) patients, which is defined as synchronous peritoneal metastasis (SPM) (1, 2). PM often has a poorer prognosis than liver or lung metastasis in CRC patients (3, 4). Patients with colorectal PM are classified into the M1c group in the eighth edition of the American Joint Committee on Cancer tumor–node–metastasis classification (5), representing a heterogeneous population of oncological prognosis.

With the increasing understanding of colorectal PM over the past decades, the treatment has changed from palliative chemotherapy to selective cytoreductive surgery (CRS) plus hyperthermic intraperitoneal chemotherapy (HIPEC) (6, 7). CRS can remove macroscopic tumors, and HIPEC can remove residual cancer cells and microscopic lesions, which significantly prolong the survival of patients (8, 9). However, studies reported considerable variations in the overall survival (OS) for CRC patients with PM due to unknown tumor heterogeneity and lack of unified treatments. Therefore, it is crucial to understand the mechanism of SPM further and predict patients’ survival accurately for better clinical decision-making.

Recent studies on basic tumor biology have shown that the tumor microenvironment (TME) plays a vital role in remodeling metastatic capacity and determining tumor prognosis (10–12). Cancer-associated fibroblasts (CAFs) are the major cellular components of the TME and have recently been regarded as critical factors to modulate the TME (13, 14). Fibroblasts and myofibroblasts are representative CAFs in fibrotic tumor stroma and are associated with tumor progression (14, 15). Their histological entities at the front of the tumor are called desmoplastic reaction (DR), which was first described in advanced rectal cancer at St. Mark’s Hospital in the UK (16). DR category is divided into three types, namely, mature, intermediate, and immature, and the prognosis deteriorates accordingly (17, 18). Studies have revealed that DR classification is associated with the prognosis of T2 CRC (19), stage II and stage III CRC (20–23), and resectable and unresectable stage IV CRC (24). In comparison, relatively few studies involved stage IV CRC. Ueno et al. found that DR classification was associated with the prognosis of resectable colorectal liver metastasis in 2014 (25). Furthermore, Ao et al. demonstrated that the DR patterns of liver and lymphatic metastases were morphologically consistent between primary and metastatic lesions in 2019 (26). In addition, Ubink et al. reported that molecular and histopathological classification of most primary tumors is consistent with corresponding metastasic tumors in colorectal cancer (27). Therefore, for CRC patients with SPM, the DR category of the primary tumor may be consistent with corresponding metastatic tumors.

However, the TME of colorectal SPM is poorly understood. DR is an embodiment of TME, whose role in SPM has not been revealed. Based on current advances, we postulate that the DR category in the primary tumor of CRC patients with SPM is associated with aggressive tumor behavior and may be a potential prognostic factor.

Therefore, this study aimed to evaluate the value of the DR category in predicting the overall survival of colorectal cancer patients with SPM who underwent CRS and to develop and validate an innovative prognostic nomogram using DR classification combined with traditional clinico-pathological parameters.

## Materials and Method

### Patients and Study Criteria

A total of 297 CRC patients with SPM were enrolled from the Sixth Affiliated Hospital of Sun Yat-sen University between July 2007 and July 2019. These patients were randomly divided into the training cohort (198 patients) and the validation cohort (99 patients) by the R software.

The inclusion criteria were patients who underwent CRS with histologically diagnosed SPM and patients with available clinicopathological data. The exclusion criteria were patients with other primary tumors.

A retrospective analysis of medical records, including surgical, pathological, and follow-up information, was conducted by the authors. Baseline clinicopathological data included sex, age at diagnosis, tumor location, tumor differentiation, tumor histology, lymph node metastasis, depth of invasion, CRS, and HIPEC. In addition, two pathologists (WD and YH) independently identified the DR classification of the primary tumor without knowing the patient’s clinical outcomes.

### Histology Categorization of DR

Hematoxylin and eosin (H&E)-stained glass slides of the primary tumor with a single longitudinal section and deepest part were obtained from the pathology department. DR was evaluated according to previous reports and histologically categorized into three categories (immature, intermediate, or mature) based on whether keloid-like collagen or myxoid stroma at the extramural of the desmoplastic front existed (16). Keloid-like collagen is characterized by bundles of hypocellular collagen with bright eosinophilic hyalinization. Myxoid stroma is an amorphous material composed of an amphoteric or slightly basophilic extracellular matrix, usually intermixed with randomly oriented keloid-like collagen (16, 17).

**Figure 1:**
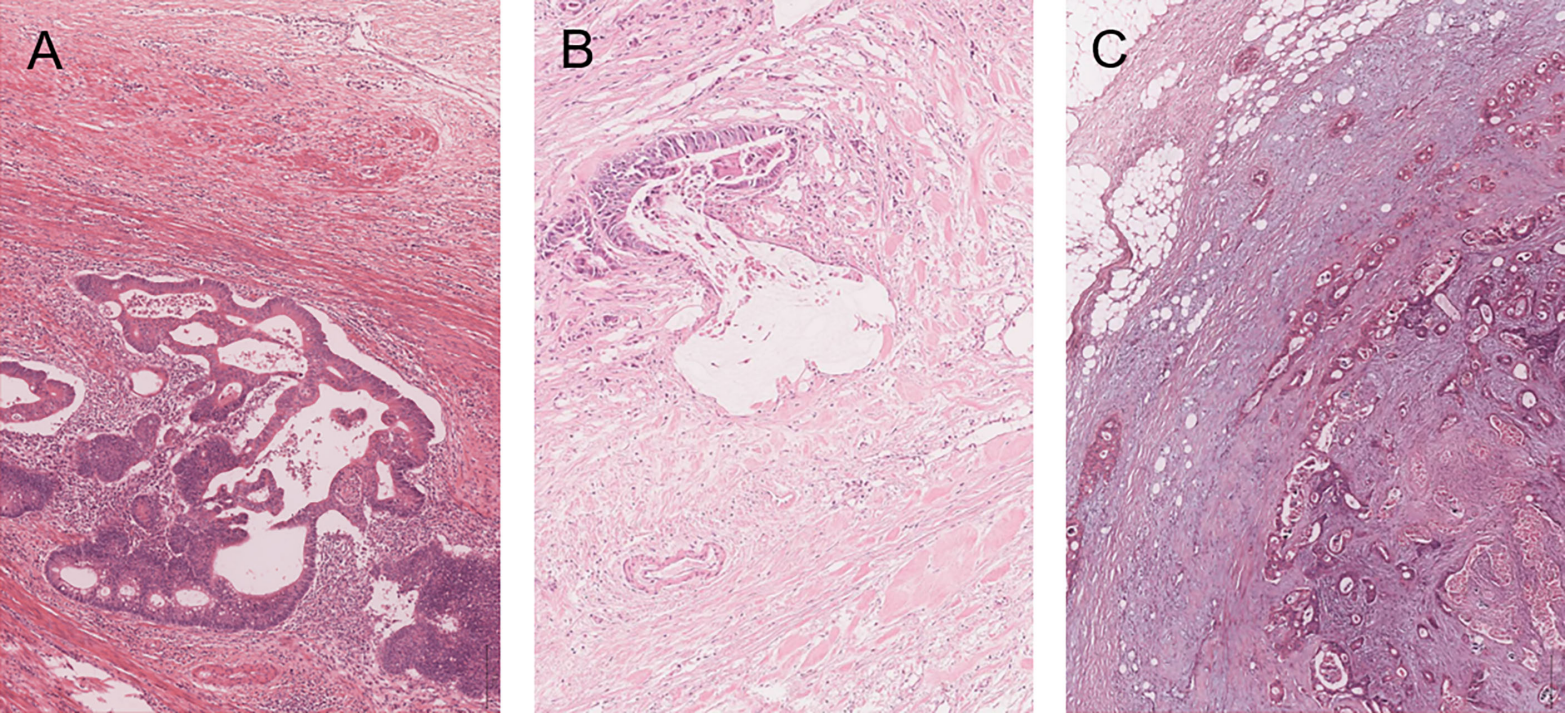
Categorization of desmoplastic reaction (DR) in the primary tumor of colorectal cancer patients with SPM. Mature DR has neither keloid-like collagen nor myxoid stroma in the fibrotic stroma and elongated collagen fibers stratified into multiple layers by fine collagen fibers **(A)**. Intermediate DR has keloid-like collagen, which is characterized by bundles of hypocellular collagen with bright eosinophilic hyalinization **(B)**. Immature DR has an amphoteric or slightly basophilic extracellular matrix that forms myxoid stroma **(C)**.

More specifically, mature DR was defined as fibrotic stroma stratified into multiple layers by fine collagen fibers without keloid-like collagen or myxoid stroma. Keloid-like collagen intermingled in the mature stroma was regarded as intermediate DR. Fibrotic stroma with myxoid stroma was designated as immature DR (**Figure 1**).

### Treatment Approaches

CRS involved removal of the primary tumor, removal of the invading organs, lymph node dissection, and/or peritonectomy, usually performed after evaluation by the multidisciplinary team (MDT). Residual lesions were evaluated by the completeness of cytoreduction score (CC score): CC0, no macroscopic peritoneal tumor remained following cytoreduction; CC1, presence of tumor nodules <2.5 mm; CC2, presence of residual disease measuring 2.5 to 2.5 cm; and CC3, presence of tumor nodules >2.5 cm, or a confluence of unresectable tumor nodules at any site within the abdomen or pelvis (28). HIPEC was performed with the closed abdomen technique. Briefly, four tubes (two for inflow of chemotherapeutic drugs and saline and two for outflow) were placed in the abdominal cavity at the end of the procedure. There were several drugs (including 5-FU, oxaliplatin, or loplatin) for HIPEC. The duration was usually at least 1 h and the fluid temperature in the abdominal cavity was kept at 42°C by a thermal perfusion device. All patients received at least two HIPEC treatments within 24 to 72 h postoperatively. Systematic chemotherapy and targeted therapy were carried out under the guidance of oncologists. Sixteen (5.4%) patients received preoperative chemotherapy and 182 (61.3%) patients received postoperative chemotherapy (adjuvant or palliative chemotherapy). For some patients, targeted therapy was added based on the results of the genetic tests. The chemotherapy regimens were mainly 5-fluorouracil-based chemotherapy, including FOLFOX, FOLFIRI, XELOX, etc. Targeted agents contained cetuximab or bevacizumab. At least three courses of continuous chemotherapy were performed for patients with chemotherapy.

### Follow-Up and Outcome

The last date of follow-up was conducted until June 20, 2021. The primary endpoint was OS, defined as the date of initial treatment (chemotherapy or surgical intervention) to the date of death or last follow-up in censored patients. Follow-up information was obtained from the hospital’s follow-up office.

### Development of the Nomogram

In the training cohort, the Kaplan–Meier method was used to generate survival curves of different variables, and the log-rank test was conducted to identify variables with *P*-values less than 0.05. Cox univariate proportional hazard regression was further used to verify the above variables. These variables with *P <*0.05 were included in Cox multivariate regression to identify independent prognostic factors. Based on the results of multivariate analysis, a nomogram was established using the R software.

### Validation of the Nomogram

Firstly, the nomogram was subjected to 1,000 bootstrap resamples for internal validation with the training cohort and for external validation with the validation cohort. The coherence of the nomogram for predicting OS between predicted and actual outcomes was evaluated by C-index. The C-index is between 0.5 and 1, with 0.5 being completely random and 1 being perfectly predictive. Furthermore, calibration plots were constructed by comparing the predicted and actual survival of 1, 2, and 3 years. Finally, the relative operating characteristic (ROC) curve was used to further verify the prediction performance in both cohorts.

### Statistical Analysis

In this study, the continuous variable age was converted to a categorical variable. The chi-square test and Fisher’s exact test were used to compare differences between categorical variables. The Kaplan–Meier curve and log-rank test were used to estimate differences in overall survival. Cox proportional hazard regression analysis was conducted to compute HR and 95% confidence intervals and to identify prognostic variables. R software (Version 4.0.3) and SPSS software (version 25.0 for Windows; Chicago, IL, USA) were used for statistical analysis. R packages of “rms,” “survival,” “foreign,” and “survivalROC” were used to construct the prognostic nomogram, calculate the C-index, and plot calibration curves and ROC curves. Decision-curve analysis (DCA) was performed with the package of “ggDCA” to evaluate the clinical practicality of the prognostic nomogram by quantifying the net benefit. A two-sided *P <*0.05 was considered to be statistically significant.

## Results

### Patients Characteristics and Overall Survival

There were no statistically significant differences in the baseline clinicopathological characteristics of the training and validation cohorts (**Table 1**). Among the 198 patients in the training cohort, 75.3% were younger than 65 years, with 122 (61.6%) men. Of the 99 patients in the validation cohort, 77.8% were younger than 65 years, with 57 (57.6%) men. The median follow-up duration (interquartile range, IQR) for all patients was 46 (29–64) months, and the median OS (IQR) for the whole cohort was 20 (10–63) months. In the training cohort, the median OS (IQR) was 22 (10–49) months and 130 patients died, and the 1-, 2-, and 3-year OS rates were 67.4%, 46.2%, and 33.7%, respectively. In the validation cohort, the median OS (IQR) was 18 (9–63) months and 68 patients died, and the 1-, 2-, and 3-year OS rates were 87.9%, 39.9%, and 30.5%, respectively.

**Table 1:** Characteristics of the patients in the training and validation cohorts.

Variable	Training cohort (*n* = 198)	Validation cohort (*n* = 99)	*P*-value
Sex			0.502
Male	122 (61.6)	57 (57.6)	
Female	76 (38.4)	42 (42.4)	
Age (years)			0.631
≤65	149 (75.3)	77 (77.8)	
>65	49 (24.7)	22 (22.2)	
Tumor location			0.756
Right side	93 (47.0)	43 (43.4)	
Left side	75 (37.9)	38 (38.4)	
Rectum	30 (15.2)	18 (18.2)	
T stage			0.869
T1–3	88 (44.4)	45 (45.5)	
T4	110 (55.6)	54 (54.5)	
Lymph node metastasis			0.492
No	28 (14.1)	17 (17.2)	
Yes	170 (85.9)	82 (82.8)	
Extraperitoneal metastasis			0.930
No	133 (67.2)	67 (67.7)	
Yes	65 (32.8)	32 (32.3)	
Histology			0.210
Adenocarcinoma	134 (67.7)	74 (74.7)	
Mucinous adenocarcinoma and signet ring cell carcinoma	64 (32.3)	25 (25.3)	
Differentiation status			0.505
Poor and undifferentiated	84 (42.4)	38 (38.4)	
Moderate and well	114 (57.6)	61 (61.6)	
CRS			0.108
CC0–1	69 (34.8)	44 (44.4)	
CC2–3	129 (65.2)	55 (55.6)	
HIPEC			0.609
No	124 (62.6)	65 (65.7)	
Yes	74 (37.4)	34 (34.3)	
Desmoplastic reaction			0.498
Mature	72 (36.4)	37 (37.4)	
Intermediate	70 (35.4)	40 (40.4)	
Immature	56 (28.3)	22 (22.2)	

### Prognostic Impact of DR Category

**Figure 2:**
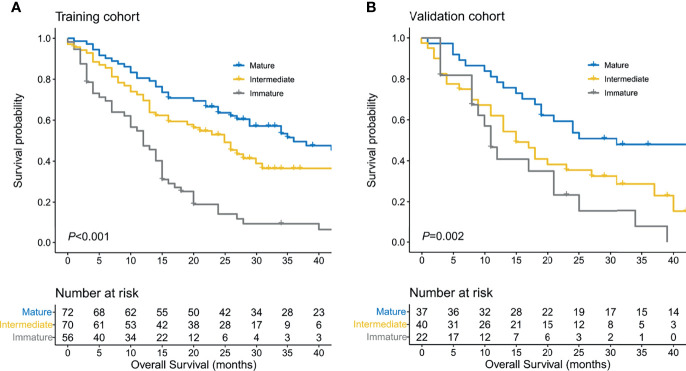
Survival estimates of the training cohort **(A)** and validation cohort **(B)** using the Kaplan–Meier method based on desmoplastic reaction (DR) categorization in the primary tumor.

In the training cohort, the DR category was classified as mature, intermediate, or immature for 72, 70, and 56 primary tumors, respectively. The Kaplan–Meier curves showed OS in the three groups (**Figure 2**). Patients with immature stroma had a worse prognosis (median OS = 36 months in mature DR, 25 months in intermediate DR, and 12 months in immature DR; *P* < 0.001, log-rank test). Similar analyses were conducted in the validation cohort. DR category was classified as mature, intermediate, or immature for 37, 40, and 22 primary tumors, respectively, in the validation cohort. The median OS was 31, 15, and 11 months, respectively (*P* = 0.002, log-rank test).

### Univariate and Multivariate Analyses in the Training Cohort

In the univariate analysis, eight variables (age > 65 years at diagnosis, T4 stage, extraperitoneal metastasis, histology of mucinous adenocarcinoma and signet ring cell carcinoma, poor differentiation, CRS, HIPEC, and DR category) were significantly associated with OS (**Table 2**). However, histology was not an independent prognostic factor when these factors above were incorporated into the multivariate analysis (**Table 2**).

**Table 2:** Univariate and multivariate analyses for overall survival (OS) by the Cox proportional hazards regression model in the training cohort.

Variable	Univariate analysis		Multivariate analysis	
	HR (95% CI)	*P-*value	HR (95% CI)	*P*-value
Sex		0.856		
Male	Reference			
Female	1.033 (0.725–1.473)			
Age (years)		0.003		
≤65	Reference		Reference	<0.001
>65	1.780 (1.217–2.603)		2.844 (1.894–4.270)	
Tumor location		0.221		
Right side	Reference			
Left side	0.719 (0.492–1.050)			
Rectum	0.807 (0.484–1.344)			
T stage		0.005		
T1–3	Reference		Reference	0.022
T4	1.664 (1.165–2.379)		1.549 (1.067–2.249)	
Lymph node metastasis		0.311		
No	Reference			
Yes	1.302 (0.781–2.172)			
Extraperitoneal metastasis		0.015		
No	Reference		Reference	0.002
Yes	1.559 (1.092–2.227)		1.855 (1.252–2.750)	
Histology		0.004		
Adenocarcinoma	Reference		Reference	0.122
Mucinous adenocarcinoma and signet ring cell carcinoma	1.678 (1.176–2.395)		1.612 (0.880–2.955)	
Differentiation status		0.002		
Poor and undifferentiated	Reference		Reference	0.001
Moderate and well	0.579 (0.409–0.818)		0.340 (0.180–0.643)	
CRS		<0.001		<0.001
CC0–1	Reference		Reference	
CC2–3	3.162 (2.096–4.769)		3.430 (2.234–5.267)	
HIPEC		0.022		0.015
No	Reference		Reference	
Yes	0.649 (0.448–0.840)		0.614 (0.414–0.910)	
Desmoplastic reaction		<0.001		
Mature	Reference		Reference	<0.001
Intermediate	1.380 (0.889–2.143)		1.671 (1.057–2.641)	
Immature	3.288 (2.140–5.052)		3.673 (2.317–5.822)	

### Construction and Validation of the Nomogram

**Figure 3:**
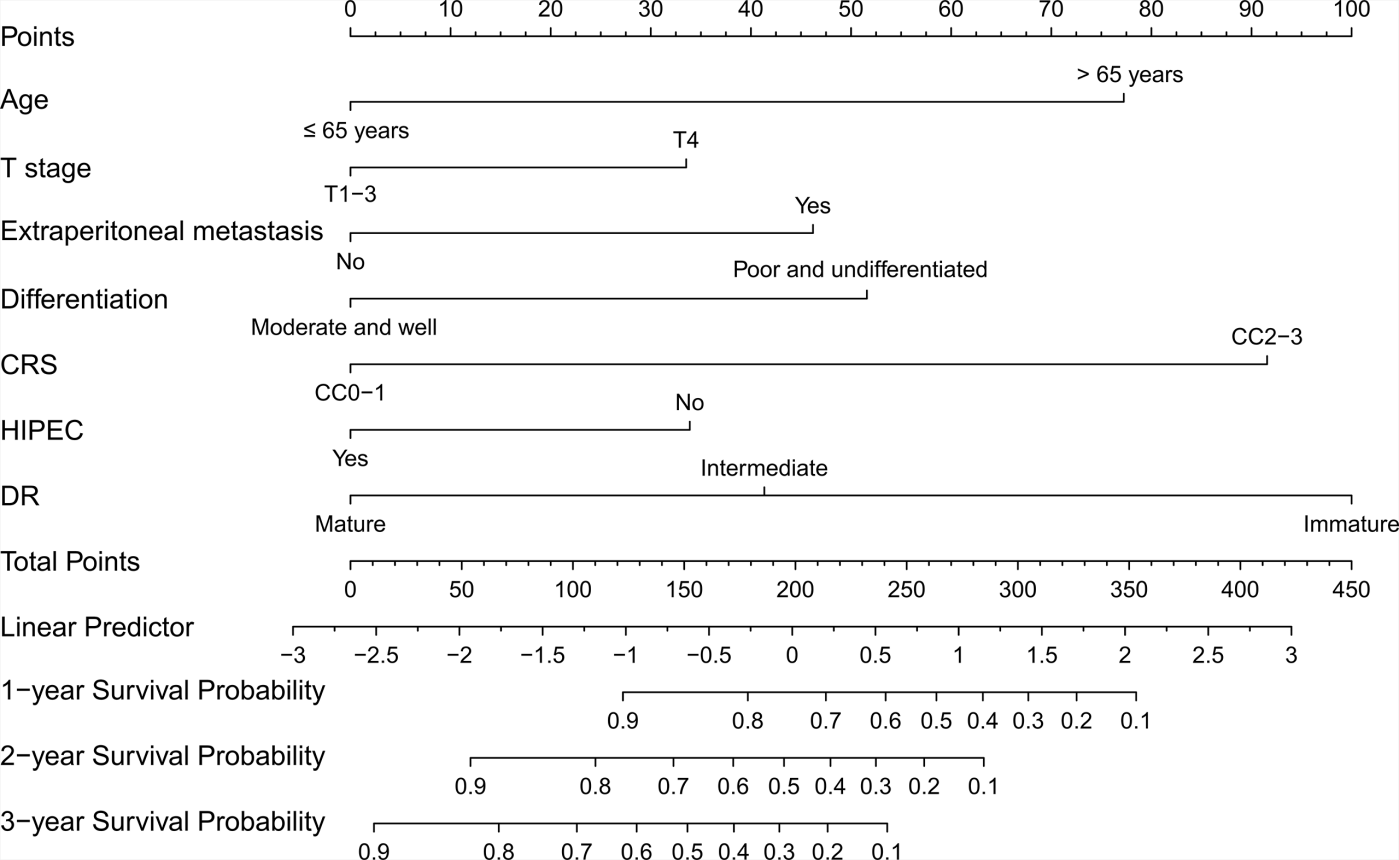
Nomogram for predicting the overall survival of colorectal cancer patients with SPM. The C-index of the nomogram is 0.773 (95% CI 0.734–0.812).

Based on the results of multivariate analysis, seven variables (age > 65 years at diagnosis, T4 stage, extraperitoneal metastasis, poor differentiation, CRS, HIPEC, and DR category) were used to construct the nomogram. This model can be used to predict the 1-, 2-, and 3-year postoperative survival probability of SPM patients treated with CRS (**Figure 3**).

**Figure 4:**
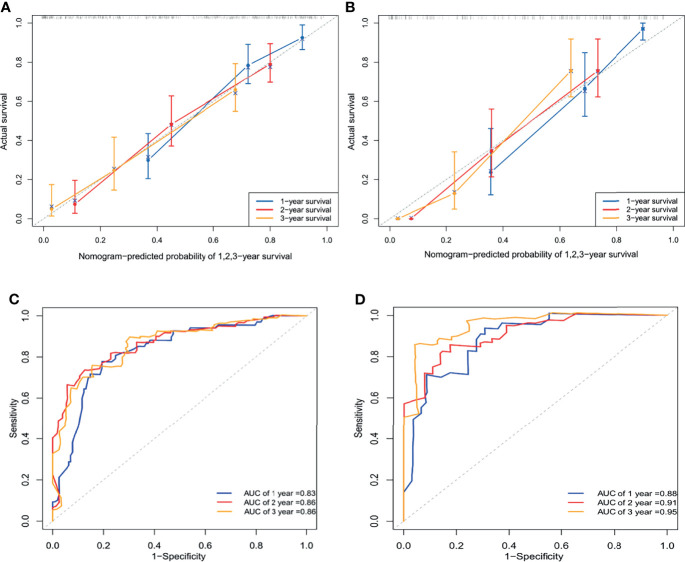
Calibration curve to validate the nomogram for 1-, 2-, and 3-year overall survival with the training cohort and its C-index was 0.773 (95% CI 0.734–0.812) **(A)**. Calibration curve to validate the nomogram for 1-, 2-, and 3-year overall survival with the validation cohort and its C-index was 0.767 (95% CI 0.708–0.826) **(B)**. ROC curve of 1-, 2-, and 3-year survival prediction in the training cohort **(C)**. ROC curve of 1-, 2-, and 3-year survival prediction in the validation cohort **(D)**. AUC, the area under the curve.

Next, C-indices were calculated for the nomogram in predicting the 1-, 2-, and 3-year OS of patients. C-indices were 0.773 (95% CI 0.734–0.812) and 0.767 (95% CI 0.708–0.826) in the training and validation cohorts, respectively. These results indicated that the model has excellent predictive ability. Furthermore, calibration plots at 1, 2, or 3 years showed good consistency between predicted survival and actual survival, either in the training cohort or the validation cohort. Similar results were further verified by the ROC curve (**Figure 4**). Besides, DCA showed better net clinical benefit of the nomogram than the model without DR classification (**Figure S1**).

## Discussion

To our knowledge, the present study is the first to reveal that fibrotic characteristics in the front of the primary tumor, known as DR classification, played an important role in predicting the OS of CRC patients with SPM. Firstly, the Kaplan–Meier method was used to analyze the prognosis of SPM patients with different DR classifications. Cox multivariate analysis revealed that DR classification could be an independent prognostic factor. Secondly we constructed and validated a prognostic nomogram to demonstrate that DR classification of the primary tumor can be a robust prognostic factor independent of traditional tumor factors in predicting overall survival.

In recent years, studies on tumor biology have shown that genes associated with poor prognosis are expressed in tumor stromal cells rather than cancer cells (29, 30). The potential for tumor growth and metastasis may depend on how tumor cells benefit by reshaping the stroma through different molecular mechanisms. Keloid-like collagen and myxoid stroma are distinct fibrotic stroma features formed by activated CAFs, although these features can also be seen in non-malignant diseases (31, 32), such as inflammatory responses to infection and benign tumors. However, the present study found that these features are mainly located in the front of the primary tumor of colorectal cancer. Our results suggested that these features were location-specific prognostic markers.

Although the mechanism of DR formation in different morphologies cannot be elucidated, it may be related to the following mechanisms. TGF-β family signaling is a possible mechanism. Keloid-like collagen bundles can be seen not only in intermediate DR but also in immature DR and are the main histological feature of scar and keloid. Compared with normal fibroblasts, fibroblasts in keloid upregulate the expression of several growth factors, including TGF-β (33). Elevated TGF-β levels have been reported to be associated with CRC recurrence (34), and non-mature DR types are associated with unfavorable survival outcomes (17). Moreover, epithelial–mesenchymal transition (EMT) is more common in malignant tumors, which is related to tumor invasion and progression (35). TGF-β can induce the activation of EMT (36). In addition, immature DR is characterized by excessive extracellular matrix deposition, including fibronectin, which affects pro-tumor functions and is associated with EMT activation (21). Therefore, it is speculated that the TGF-β signaling pathway activates EMT as a possible mechanism for non-mature DR formation.

Histological features of unfavorable DR classifications also include reduced immune cell infiltration (20, 21) and reduced microvascular formation (21). Ozdemir et al. observed that myofibroblast depletion in pancreatic cancer led to immunosuppression and increased tumor aggressiveness in transgenic mice using deleted αSMA+ myofibroblasts (37). Therefore, it can be speculated that CAFs are involved in tumor stroma remodeling and immunosuppression. On the other hand, mature DR is characterized by thin, multilayered, mature collagen and neatly arranged fibroblasts, which can encapsulate the tumor nests and inhibit metastasis. These results suggest that different CAF subgroups may be involved in forming different DR types, and future studies need to confirm this conjecture further.

DR classification as a prognostic factor has been validated not only in different stages of colorectal cancer but also in pancreatic ductal carcinoma (38), cervical squamous cell carcinoma (39), intrahepatic cholangiocarcinoma (40), and esophageal squamous cell carcinoma (41). In particular, a recent retrospective phase III clinical trial concluded that DR might be a valuable prognostic indicator to identify patients who will benefit from postoperative chemotherapy in stage II colorectal cancer (22). This result further confirms the prognostic value of DR. The prospective phase III clinical trial (JCOG1805) launched in 2020 in Japan is expected to further clarify the role of DR in stage II CRC patients at high risk of developing recurrence according to T stage and three selected pathological factors. In a word, we found that DR classifications could be used to predict postoperative OS of CRC patients with SPM, which is in accordance with expectation. Of course, further multicenter, prospective validation is needed.

This study has some strengths. Firstly, this is the first study to reveal that DR category is associated with OS of CRC patients with SPM. It may guide clinical decision-making and provide a new perspective for further understanding the mechanism of the occurrence and progression of colorectal PM. Secondly, the nomogram in this study may be superior to the peritoneal surface disease severity score (PSDSS) and the colorectal peritoneal metastases prognostic surgical score (COMPASS) in terms of prediction of OS for patients with colorectal peritoneal metastasis, which did not include the factor of tumor stroma (42, 43). Geert et al. performed an external validation of the PSDSS, showing a Harrell’s C statistic of 0.62, and further developed the COMPASS with a Harrell’s C statistic of 0.72, while the nomogram in the present study showed C-indices of 0.773 (95% CI 0.734-0.812) in the training group and 0.767 (95% CI 0.708–0.826) in the validation group. Thirdly, in terms of the clinical significance of the nomogram model, it may help select patients who could benefit from CRS and HIPEC preoperatively by endoscopic biopsy and provide a reference for clinical decision-making. For example, aggressive CRS may not be necessary for patients who are not expected to achieve complete CRS and with adverse DR classification. Modern palliative chemotherapy may be a better selection for these patients.

This study also has some limitations. First of all, this is a retrospective study, and the single-center data used in this study may have some bias. However, it is the largest cohort reported to determine the prognostic value of DR classification in CRC patients with SPM. Although we conducted a validation with data of random allocation, prospective, multicenter studies are needed for further validation. Secondly, some of the SPM patients enrolled in this study failed to achieve complete CRS. Limited by sample size, the effect of DR classification on relapse-free survival in CRC patients with SPM cannot be further clarified. Thirdly, due to no CRC cases with metachronous peritoneal metastasis being included in this study, we apologize for not presenting the prognostic value of desmoplastic reaction in patients with metachronous peritoneal metastasis. Future studies to validate the prognostic value of desmoplastic reaction in patients with metachronous peritoneal metastasis are expected. Finally, the current classification of DR relies on the artificial classification of pathologists. If collagen features can be extracted and quantified, the prognosis may be better predicted.

## Conclusions

In conclusion, we developed and validated an innovative nomogram to predict the OS of CRC patients with SPM based on fibrotic stroma classification in the primary tumor. This model can provide a vital prognosis-predicting tool for these patients.

## Data Availability Statement

The raw data supporting the conclusions of this article will be made available by the authors, without undue reservation.

## Ethics Statement

The studies involving human participants were reviewed and approved by the Institutional Review Board of The Sixth Affiliated Hospital of Sun Yat-sen University (No. 2020ZSLYEC–109). The ethics committee waived the requirement of written informed consent for participation.

## Author Contributions

XQ, KY, DC, and HW contributed to the conception and design of the study. XQ, MZ, WD, YH, ZC, JC, and XC contributed to data acquisition. XQ, MZ, and WD performed the statistical analysis. XQ wrote the first draft of the manuscript. MZ and WD wrote sections of the manuscript. All authors contributed to manuscript revision and read and approved the submitted version.

## Funding

This study was supported by Sun Yat-sen University Clinical Research 5010 Programs (grant numbers 2017008 and 2019021).

## Conflict of Interest

The authors declare that the research was conducted in the absence of any commercial or financial relationships that could be construed as a potential conflict of interest.

The handling editor declared a shared parent affiliation with several of the authors XQ, WD, YH, ZC, KY, HW at the time of review.

## Publisher’s Note

All claims expressed in this article are solely those of the authors and do not necessarily represent those of their affiliated organizations, or those of the publisher, the editors and the reviewers. Any product that may be evaluated in this article, or claim that may be made by its manufacturer, is not guaranteed or endorsed by the publisher.

## Supplementary Material

Supplementary Figure 1 Decision curve analyses (DCA) of nomogram prediction model and model without DR category for 1,2,3-year OS in the training cohort **(A-C)**, and the validation cohort **(D-F)**.
