# Supplementary material for: Development and Validation of a Novel Prognostic Nomogram Combined With Desmoplastic Reaction for Synchronous Colorectal Peritoneal Metastasis
Source: Front Oncol. 2022 Mar 11;12:826830. doi: 10.3389/fonc.2022.826830 (PMC8963183; doi:10.3389/fonc.2022.826830)
Supplement: Supplementary Figure 1 — Decision curve analyses (DCA) of nomogram prediction model and model without DR category for 1,2,3-year OS in the training cohort (A-C), and the validation cohort (D-F). [file Presentation_1.pptx]

## Slide 1
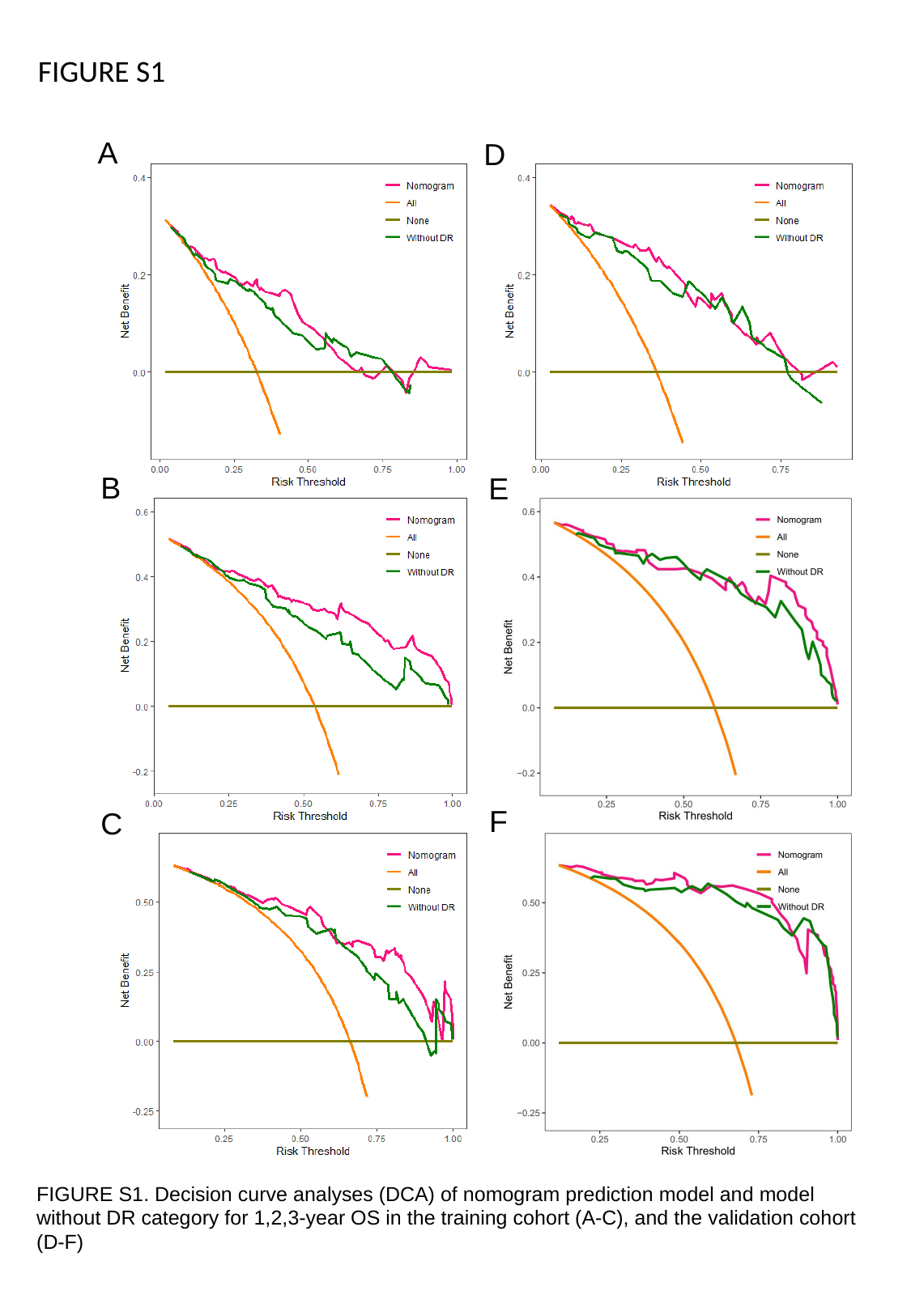

FIGURE S1
A
D
B
E
F
C
FIGURE S1. Decision curve analyses (DCA) of nomogram prediction model and model without DR category for 1,2,3-year OS in the training cohort (A-C), and the validation cohort (D-F)
